# Nursing Process in Post Tonsillectomy Pain Diagnosis: A Systematic Review

**DOI:** 10.5539/gjhs.v7n1p180

**Published:** 2014-08-22

**Authors:** Fateme Soleymanifard, Seyyed Mohamad Khademolhoseyni, Jamile Mokhtari Nouri

**Affiliations:** 1 Student of MSc. Nursing, Behavioral Sciences Research Center (BSRC), School of Nursing, Baqiyatallah University of Medical Sciences, Tehran, Iran; 2 Department of Pediatrics, School of Nursing, Baqiyatallah University of Medical Sciences, Tehran, Iran; 3 Department of Management, School of Nursing, Baqiyatallah University of Medical Sciences, Tehran, Iran

**Keywords:** nursing care, pain, pediatric, tonsillectomy

## Abstract

**Objective:**

Tonsillectomy is the most common surgery in the field of ENT. Pain is the most common post tonsillectomy complaint. Considering the importance of nursing cares in relieving post-surgery pain in general and post-tonsillectomy pain in particular, this study is conducted with the aim of presenting nursing process in post tonsillectomy pain diagnosis for decreasing loss of appropriate opportunities in nursing cares and achieving appropriate results in taking care of the patients.

**Methods:**

This study is a targeted systematic review focusing on “effective nursing measures in relieving children’s post tonsillectomy pain”. The main stages of searching strategy included searching in electronic sources of Latin databases; Pub Med, Science Direct, and EMBASE and Persian databases; SID, Iran medex, ISC to find published articles from 2009 to 2014. In the end, final synthesis was done on eight articles in English.

**Findings:**

Effective nursing measurements for relieving post tonsillectomy pain include: decreasing children’s anxiety through children and their families’ psychological preparation by nurses and other caregivers, using cold compress to reduce neck and jaw pain, presenting distraction techniques, offering fluids and cold foods immediately in the period after surgery, creating a comfortable environment for the children, avoiding too much of talking and adequate sleep.

**Conclusion:**

It is recommended to the nursing managers and nurses to perform cares achieved from this systematic review to achieve appropriate results in relieving post tonsillectomy pain.

## 1. Introduction

Adenoids or throat tonsils are consisted of lymphatic tissue and are closeto the nose pharynx posterior wall. Beta hemolytic-streptococci group A is the most common cause of tonsillitis. Enlarged adenoids may cause mouth breathing, earache, colds, recurrent bronchitis, halitosis, difficulty in swallowing, sound disorder and noisy breathing. Infected adenoids are frequently along with acute tonsillitis. Bacterial infection with penicillin prescription is the front-line drug treatments once antibiotics does not influence tonsillitis (Smeltzer, 2012). Tonsillectomy is three thousand years old (Fida & Sendi, 2013). Tonsillectomy with or without Adenoidectomy is still the most common surgery in the field of ENT (Faramarzi & Heydari, 2010). There are several serious post tonsillectomy complications; pain is the most common post tonsillectomy complaint (Smith et al., 2009). Pain is a subjective experience that its presence or absence can’t be proved (Wright & Schutte, 2014). According to the definition of pain international association, pain is an unpleasant sensory and emotionally experience that is due to actual or probable tissue damage (Harden et al., 2010). Although, no pain can be actually monitored, nowadays they are considered as the fifth vital signs in clinical cares. Children cannot express pain verbally until achieving speaking ability completely and it is necessary to use concrete tools for measuring their pain (Sadeghi et al., 2012).

Pain can be somatic or sensory and it is counted as the care principles in nursing (Alavi et al., 2013). Currently, pain is counted as the cause of more than 80% of medical appointments in America, because it has influenced more than 50 million Americans and also healthcare costs. Health and treatment costs and damages due to do that in compare with annual national production is estimated more than seventy billion dollars. Considering the above astronomical numbers, it should be admitted that incomputable patients’ suffering is simply ignored. Despite new treatment methods and different kinds of surgeries and drugs, pain is still the basic problem of the medical society (xa.yimg.com, 2011). When we start talking about issues in pediatrics, the first important point is that children are not adults with smaller size, but in many cases, features related to their body structure and as the result their problems and diseases are completely different with adults (Ahmadi, 2013). Pain management is one of the important rights of children and it is one of the priorities of treatment. Relieving pain prevents adverse consequences and serious complications and it promises normal growth and development of the next generation. Nurses are among the important people who have the highest relationship with the children suffering from pain and they can prevent future problems through assessing and treating children’s pain (Namnabati et al., 2008).

Post tonsillectomy effective pain management is one of the important aims. There is a direct relationship between pain and problems such as; inadequate consumption of fluids, dehydration and need for more medical interventions (Windfuhr, 2013). Most of the studies regarding post tonsillectomy pain control are pharmacological studies and they are actually about comparison of the effect of two drugs in relieving pain or drugs complications. There are also some studies regarding comparison of the surgical methods and their relationship with post-surgery pain. Despite the importance of nursing cares in relieving post-surgery pain in general and children’s post tonsillectomy pain in particular, lack of coherent studies for the nurses to survey and assess different mental, psychological and physical dimensions of a child and to perform some measures for relieving pain can be observed. This study is done for decreasing loss of appropriate opportunities in nursing cares and achieving appropriate results in children’s nursing cares after tonsillectomy with the aim of presenting nursing effective measurements in relieving post tonsillectomy pain.

## 2. Methods

This study is a targeted systematic review focusing on “effective nursing measurements in relieving children’s post tonsillectomy pain”.

### 2.1 Selection Criteria

All the articles that evaluated nursing cares in relieving pain of the children undergoing tonsillectomy from 2009 to 2012 were assessed with the following priorities that include: “1. a systematic review and RCT articles 2. Articles that their full texts were in access and 3. Articles that were written in Persian and English”.

Searching electronic information sources was done in the selected databases during a certain time interval (February 2014). For preventing bias, the research was done by the two researchers simultaneously and independently.

### 2.2 Data Extraction

**Graph 1:**
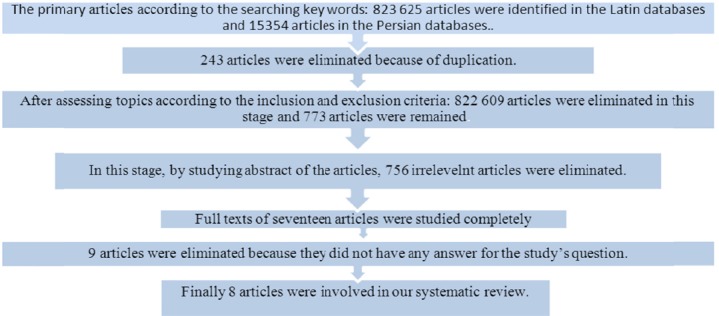
Diagram of entry process of the primary studies into the final synthesis and their exiting process from that

In the first stage, key words were selected by the MESH explorer. Key words including: nursing care, tonsillectomy, pain and pediatric were searched in the Latin databases: Cochran, Pub Med, Science Direct and EMBASE and the same key words in Persian were searched in the Persian databases: ISC, SID and Iran Medex. At first searching key words was done in its single form in every database and in the end 823625 articles were identified. Based on different databases, some changes were madein the searching method. Combination of appropriate key words was used with the aim of conducting a sensitive research in the databases. The search was done in the advanced search of Pub Med and Science Direct databases. Considering inclusion and exclusion criteria, after performing qualitative study, 8 articles had the inclusion criteria. It should be mentioned that in the first stage of screening, the topics and in the second stage the abstract of the articles were assessed. Qualitative evaluation of the articles were also assessed by two browsers of the group members and in the case of disagreement, it was referred to the third browser. Graph 1 shows entry process of the primary studies into the final synthesis and their exiting process from that.

## 3. Results

In the end, after searching, screening and qualitative evaluation of the studies during systematic review, final synthesis was done on eight English articles (Table 1).

**Table 1:** Articles features

Number of the article	Writer	The year of the study	Type of the study	Sample size	Samples’ age	The place of the study	Reference number	Magazine	The year of publishing article	
1	Jacqueline D	2009	A Prospective Audit	All the children who were admitted in the recovery of the hospital in a 7-day course in July 2007	1-18 years old	British children’s hospital Columbia	13	Aorn Journal	2009
2	SeijaKlemetti	2009	prospective randomized study	4—10 years	n = 134	Turkey	21	International Journal of Pediatric Otorhinolaryngology	2009
3	Kristen Kenney	2013	Systematic review	Literature search using EBSCO Host (Cinahl, Medline), Google Scholar	_	Boston Children’s Hospital	18	Journal of PeriAnesthesia Nursing	2013
4	Sylvester, D.C	2009	*retrospective descr*iptive design	5-10 years	N=400	Hospital of Philadelphia	16	Journal of PeriAnesthesia Nursing	2013
5	Sylvester, D.C	February 2010 to February 2011	A single-blinded, randomized, controlled trial	Children aged 2–12 undergoing tonsillectomy	Ninety-two	in the Department of Otolaryngology at Leeds General Infirmary	23	Clinical Otolaryngology	2011
6	Dekeisha Howard	2012	An integrative review through the perioperative and home experience	evidence published between 2005 and 2012 databases searched included Pub Med, MEDLINE, and CINAHL	_	duPont Hospital for Children, Wilmington, Delaware	14	Journal for Specialists in Pediatric Nursing	2013
7	Kimberly A.	2013	Evidence-based recommendations for prevention and management	children ages two to 10	_	USA	17	AJN	2014
8	Kimberly A.	2012	A descriptive feasibility study	ages 3 to 5 years	N=47	Central California	20	NIH Public Access Author Manuscript	2013

Results achieved from data analysis indicate that nursing process for relieving pain due to surgical manipulation, head and neck spasms and cutting the operative site in tonsillectomy includes (Tables 2, 3, 4 & 5).

**Table 2:** Nursing diagnosis

Nursing diagnosis
Pain(NANDA)
Related to: a) Surgery manipulation. b) Head and neck muscles spasms c) cutting the surgical site

Evaluation criteria
Vital signs, sleep patterns, body and face movements, mood of patient and family, interacting with others, verbal expression.

**Table 3:** Nursing assessment for relieving children’s post- tonsillectomy pain

Nursing assessment
Assessing all the patients regarding the presence of pain [13, 14].
Assessing intensity and quality of pain (Trudeau et al., 2009) based on FLACC scale in the children less than three years old (Voepel-Lewis et al., 2010) and LAPS self-report scale for the older children (Tomkinson et al., 2012).
Assessing patients regarding signs of inadequate pain relief or drugs complications such as: nausea and vomiting, dizziness, drowsiness… (Sutters & Isaacson, 2014).
Assessing pain-related behaviors and diagnosing them by stressful behaviors especially in the children that do not have the ability of verbal communication (Trudeau et al., 2009).

**Table 4:** Nursing measurements for relieving children’s post- tonsillectomy pain

Nursing measurements
1. Giving combined analgesics during surgery and in the phase one of recovery (Scalford et al., 2013).
2. Combination of pain management methods and improvement of performance and reforming programs of the institute (Trudeau et al., 2009).
3. Decreasing children’s anxiety through psychological preparation of the children and their families by the nurses and other caregivers (Trudeau et al., 2009, Howard et al., 2014).
4. Using cold compress for relieving neck and jaw pain (Howard et al., 2014; Sutters & Isaacson, 2014; Kenney & Gropman, 2013), warm compress for relieving earache and chewing gum for reducing muscle spasm (Sutters & Isaacson, 2014).
5. Providing distraction techniques (cognitive-behavioral methods for pain control such as: music, pictures, watching films and computer games) (Howard et al., 2014; Sutters & Isaacson, 2014).
6. Performing planned analgesic regimen (every six hours) for the first three days after tonsillectomy surgery (Sutters & Isaacson, 2014; Howard et al., 2014; Sutters et al., 2012).
7. Being preoperative short-term NPO (encouraging hydration before being NPO for doing surgery) (Klemetti et al., 2009).
8. Giving humidified oxygen to the patients at the rate of 2-3 liters per minute (Kenney & Gropman, 2013).
9. Advising advance diet to the patient according to his/her tolerance level (Kenney & Gropman, 2013).
10. Providing some conditions for the parents’ presence at the time of induction of anesthesia and in recovery (Howard et al., 2014; Sutters & Isaacson, 2014; Scalford et al., 2013).
11. Appropriate communication and cooperation of the staffs and family, continuing this cooperation after discharge and improving family’s education (Howard et al., 2014; Sutters & Isaacson, 2014; Scalford et al., 2013).
12. Offering fluids, cold foods and soft foods immediately in the postoperative period (Howard et al., 2014; Sutters & Isaacson, 2014; Sylvester et al., 2011).
13. advising a half-sitting position after surgery, creating a comfortable environment for the child and avoiding too much of talking and adequate sleep(Howard et al., 2014; Sutters & Isaacson, 2014).
14. Encouraging and not forcing use of fluids (Howard et al., 2014, Sutters & Isaacson, 2014).
15. Using Ibuprofen for relieving post-surgery pain is not dangerous (Sutters & Isaacson, 2014).
16. Advising parents to hold younger children in their arms and spend time for taking care of them (Sutters & Isaacson, 2014).
17. To not discharge the child from the hospital until satisfied pain control and complete analgesia (Trudeau et al., 2009).

**Table 5:** Nursing education for relieving children’s post- tonsillectomy pain

Nursing educations
1. Correction of false beliefs about this issue that using analgesic drugs is effective and safe (Sutters & Isaacson, 2014).
2. Giving preliminary information regarding measurements before the surgery, the surgery itself, reassuring the patient that the surgery is not dangerous (Sutters & Isaacson, 2014; Trudeau et al., 2009; Howard et al., 2014).
3. Presenting educational pamphlets and information about surgery, postoperative cares and the way of using analgesics (Howard et al., 2014).

Although pain cannot be monitored in its real form, nowadays, it is counted as the fifth vital signs in children’s clinical cares. Children cannot express pain verbally until their complete achievement of speaking ability sousing concrete tools for measuring children’s pain is necessary (Sadeghi et al., 2012). Pain management is one of the important rights of children and it is one of the treatment priorities. Relieving pain prevents adverse consequences and serious complications and promises normal growth and development of the future generation. Nurses are among the important groups of people that have the highest relationship with the children suffering from pain and they can prevent future problems through assessing and treating children’s pain (Namnabati et al., 2008).

Studies about pain intensity patterns and using analgesic drugs in the hospital help development of information and correction of post-tonsillectomy pain management.

Surgery and anesthesia cause remarkable anxiety and neurosis in children and their parents and it is clear that decreasing their anxiety and making them relaxed are very important since it prevents unpleasant mental consequences. Decreasing children’s anxiety leads to improvement of post-surgery results. Nowadays, there are some evidences confirming the effect of education on controlling pain.

Topical or systemic application of cold therapy has been used since hundreds years ago. Evidences show that in the presence of coldness, tissue metabolism will be decreased and also inflammation and pain can be controlled.

Pain causes muscles cramp and then muscles blood flow is reduced and they receive less oxygen and nutrients and this cycle continues. When the painful area of the body is heated, actually blood flow is increased in that area and the pain cycle is broken.

Agency for Health Care Policy and Research (AHCPR) states that; effective treatment for pain includes using pharmacological and non-pharmacological methods. Behavior therapy is among one of the most important non-pharmacological interventions of relieving pain and distraction methods is one of them. In this method, person’s attention from a painful stimulus will be distracted to pleasant stimulus and totally it decreases perceiving pain. This method, in addition to having less complication in compare with pharmacological methods or even not having any complications at all is also less costly. Also it does not have any harmful physical and psychological effects on the children, it is easy to use and most importantly it is among the independent nursing measurements and it is very attractive for young children (Shaban et al., 2006).

Humidified oxygen prevents mucous membranes drying and makes evacuation of secretions in the lungs easier.

Beliefs in pain reliving have remarkable effects on nurses’ performance. Nurses believe that prescribing opioids leads to drug dependence and it has some complications such as; respiratory depression, lethargy, lack of patient’s cooperation in getting down from the bed. It should be mentioned that physical dependence, tolerance and addiction are very different. Actually it can be said that physical dependence and tolerance are involuntary physical responses to some drugs such as opioids and if a patient’s pain is controlled by enough opioids, the patient asks for drug again, while in drug addiction, the patient tries a lot to achieve drugs and in the case of discontinuation of the drug, he/she is willing to use opioids. Studies showed that less than one percent of the patients suffer from drug addiction because of being under treatment by opioids (SM, 2012).

## 4. Discussion

The present study is conducted with the aim of nursing process in relieving children’s post-tonsillectomy pain. Findings of the present study which are pointed out in most of the selected articles include: decreasing children’s anxiety with psychological preparation of the children and their families by nurses and other caregivers is effective in decreasing post-surgery pain. Surgery anxiety may influence pain intensity of the surgery; in addition parents’ anxiety can be transmitted to the children. There are many studies that confirmed this instruction; for example in the study of Klemetti et al. (2010), it is emphasized on writing information, face-to-face counseling about being NPO, active nutrition after surgery and giving information about surgery for decreasing parents and children’s anxiety (Klemetti et al., 2010). Also in the study of Sarny et al. (2012) and Baugh (2011), it is pointed out to the effect of parents’ education on post-surgery pain management in the house (Sarny et al., 2012; Baugh et al., 2011). Results of other studies indicate the positive effect of education on deceasing post-surgery anxiety (Guo et al., 2012). Using cold compress for relieving neck and jaw pain is also one of the instructions achieved from the results of most of the studies of this systematic review; studies of Rotenberg (2013) and Shin (2014) confirm this issue. There have been few studies conducted in supporting ice collar program in post-tonsillectomy pain relief and there are many studies in supporting ice therapy in orthopedic surgeries.

The third finding of the present study is presenting distraction techniques for decreasing post- tonsillectomy pain. The results of some studies such as Helgadottir (2013), Fernandez (2010), Agostini (2013), Fortier (2011) are in consistent with this finding. Advising to use fluids and cold foods immediately in the period after surgery that in the study of Belli (2009), using cold fluids is confirmed as a safe method for post-tonsillectomy pain relief and advising to create a comfortable environment for the children and to avoid too much of talking and adequate sleep are among other results of this study and the results achieved fromthe study of Sutters (2010) is the same.

## 5. Conclusion

Lack of specific and up-to-date nursing instructions and their low quality show the necessity of designing evidence-based and specific instructions with high quality in different parts. Designing nursing cares instructions for promoting health services, considering patients’ rights and creating uniformity in presenting health policies especially in children make the cares valuable and correct the results. So it is recommended to the nursing managers and nurses to perform cares achieved from this systematic review to achieve appropriate results regarding post-operative pain relief.
